# Advances and Limitations of Next Generation Sequencing in Animal Diet Analysis

**DOI:** 10.3390/genes12121854

**Published:** 2021-11-23

**Authors:** Gang Liu, Shumiao Zhang, Xinsheng Zhao, Chao Li, Minghao Gong

**Affiliations:** 1 Key Laboratory of Wetland Ecological Function and Restoration in Beijing City, Wetland Research Institute of Chinese Academy of Forestry Sciences, Beijing 100091, China; gangl@caf.ac.cn (G.L.); surezx4@163.com (X.Z.); 2016011187@nwafu.edu.cn (C.L.); 2 Beijing Milu Ecological Research Center, Beijing 100076, China; shumiaozhang@126.com

**Keywords:** next generation sequencing (NGS), diet analysis, DNA metabarcoding, trophic link, foraging behavior, nutrigenomics

## Abstract

Diet analysis is a critical content of animal ecology and the diet analysis methods have been constantly improving and updating. Contrary to traditional methods of high labor intensity and low resolution, the next generation sequencing (NGS) approach has been suggested as a promising tool for dietary studies, which greatly improves the efficiency and broadens the application range. Here we present a framework of adopting NGS and DNA metabarcoding into diet analysis, and discuss the application in aspects of prey taxa composition and structure, intra-specific and inter-specific trophic links, and the effects of animal feeding on environmental changes. Yet, the generation of NGS-based diet data and subsequent analyses and interpretations are still challenging with several factors, making it possible still not as widely used as might be expected. We suggest that NGS-based diet methods must be furthered, analytical pipelines should be developed. More application perspectives, including nutrient geometry, metagenomics and nutrigenomics, need to be incorporated to encourage more ecologists to infer novel insights on they work.

## 1. Introduction to Diet Analysis

What an animal eats is perhaps the most ecologically important background information we can understand the species’ nutrition ecology [1,2], and diet analysis is one of the important contents of animal ecology [3,4]. It is the prerequisite for evaluating the host health, understanding the relationship between animal and environment, exploring predator-prey dynamics, uncovering trophic interactions, explaining behavioral plasticity and even faciliating pest management [5,6,7,8,9,10]. It also benefits constructing habitat selection and utilization models, determining foraging strategies and nutrient flows, assessing species’ survival status and ecosystem function, discovering the mechanistic processes behind complex food web dynamics and other hot issues [11,12]. How to accurately and precisely identify the diet compositions and proportions of different prey items remains a challenge before introducing molecular techniques. It is very important to have reliable dietary data before exploring biological and evolutionary questions involved with food intake [13].

Dietary intake is relatively very difficult to be measured reliably in humans because approaches of diet analysis typically rely on self-reporting, which can be incomplete and biased [14]. Traditional diet analysis methods include direct observation of foraging behavior, the cafeteria diet, microscopic identification of prey remains in fecal and stomach contents [15,16]. The operation of field behavior observation is poor and the results are qualitatively descriptive [15], and the cafeteria diet method is more suitable for studying animal diet preferences in captive environment [17]. The stomach contents analysis is a destructive approach and clearly less acceptable in mammalian studies [18]. Microscopic analysis has a high requirement on the microscopic identification technology and the work is labor intensive, and it has low resolution on diet items with similar micro-morphological tissues [19]. Both methods of plant alkane fingerprint and near-infrared reflectance spectroscopy are mainly used in the nutrient research of herbivores, but cannot determine the diet composition [20]. Stable isotope analysis has advantages in determining the structure of food webs and analyzing energy flows, however, it still is difficult to investigate the fine-scale diet patterns often sought in food web studies [21,22].

The molecular-based analysis of animal diets has recently become popular, as they confer high resolution and accuracy, which is mainly achieved through the cloning sequencing or the next generation sequencing (NGS) on the amplification of prey DNA in dietary samples [23,24]. Both approaches need to be combined with DNA metabarcoding using general or group-specific primers. The cloning approach has more obvious advantages than non-DNA based diet analysis methods, but has its own limitations, for example, sequencing more clones will greatly increase the workload and the cost, but the effect may not necessarily be improved [25,26,27]. With the development of NGS, this technology is gradually extended to diet analysis, and its unique advantages make relevant dietary studies emerge, covering mammals, birds, amphibians, fish and even invertebrates [28,29,30]. However, applying NGS into diet analysis has not been paid more attention as conservation genomics and ecological metagenomics, and it is still not as widely used as might be expected [31]. In this review, we present a framework of adopting NGS into diet analysis, and discuss the application in aspects of diet composition, intraspecific and interspecific trophic links, and the relationship between food resources and habitat or behavior. We suggest that NGS based diet methods must be furthered, analytical pipelines should be developed, and more application perspectives need be incorporated to encourage more ecologists to infer novel insights on they work.

## 2. Conceptual Framework of Diet Analysis Using NGS

**Figure 1:**
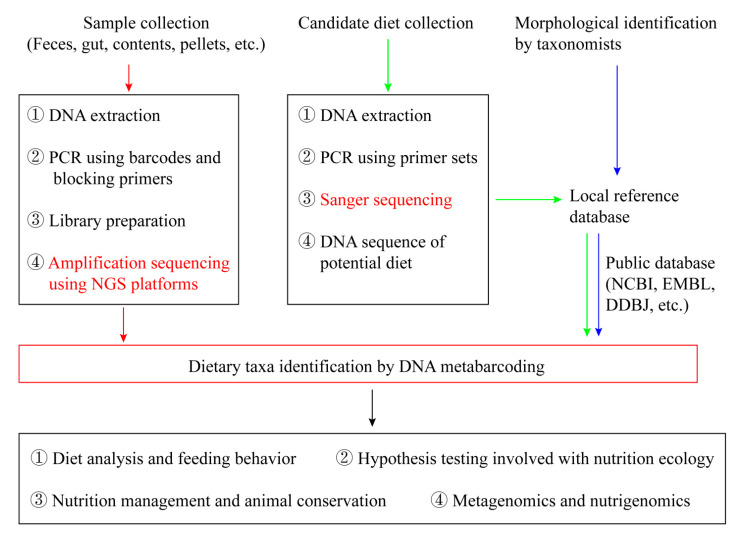
An overview of conceptual framework of diet analysis using next-generation sequencing (NGS).

The overall framework of analyzing animals’ diet based on NGS is: Collect samples (faeces or gut contents) used for prey DNA extraction; Extract prey DNA in animal pellets and remains; Select the corresponding DNA barcodes with both high universality and high resolution; Construct reference databases from potential dietary species; Conduct PCR amplification on extracted DNA; Sequence the PCR products using NGS platforms; Blast NGS generated DNA sequences with the constructed DNA barcode database consisting of local potential food resources and/or the public database; Identify food taxa according to the sequence coverage and similarity (Figure 1).

### 2.1. Sample Collection and DNA Extraction

At present, the vast majority of dietary studies applying NGS use feces as the sample, because fecal samples contain the undigested feed materials, and are easily collected. However, stomach contents are applied for studying the diet of rodents, locust and fish [8,32,33]. In some avian studies, pellets are also used as complementary samples [30]. Non-invasive sampling of feces is particularly suitable for studying animals, especially when monitoring the diet for a long time. The freshness of feces is the key to determine the quality of fecal DNA, which can directly affect the performance of DNA extraction, PCR and sequencing. The quality of fecal DNA is also related to the sampling part of feces. Sampling and mixing the center, the middle and the out layer of feces can significantly improve the detection rate of prey DNA, especially for the rare item that animals consume less frequently [34,35,36].

The effects of sample preservation methods and DNA extraction methods on DNA quality have been well studied in conservation genetics [37], but its effect on diet analysis has rarely explored. In NGS diet analysis, common preservation methods include silica-gel drying (rodents [38]; brown bear [39]), buffer solution (lizard [40]), ethanol (bat [41]), freezing (bat [28]; lizard [40]; seal [42]; great bustard [43]), etc. There are also two-step preservation methods, such as ethanol and cryopreservation (bat [44]) and ethanol and silica-gel preservation (leopard cat [45]). Researchers also need to consider the feasibility of conservation methods and the convenience of transporting into consideration.

The DNA extraction method mostly adopts the more commonly commercial kit, and also some researchers select the tissue DNA kit according to the feeding habits difference of the targeted species [46]. Most studies use QIAamp DNA Stool Mini Kit, but there are exceptions. The inhibitex in QIAamp DNA Stool Mini Kit contains the potato adsorbent, which may be mixed in DNA extraction, and it may make potato appear in the diet [47]. MoBio, Epicentre, and Qiagen’s fecal DNA extraction kits have a poorer effect than CTAB extraction method in analyzing diet of Corvus corone [48], but it may depend on the predator. For some species, the extraction effectiveness of QIAamp DNA Stool Mini Kit is significantly lower than Zymo Soil/Fecal DNA MiniPrep Kit [49]. Therefore, it is crucial to optimize the key step of fecal DNA extraction, to efficiently yield trace amounts of prey DNA while simultaneously minimizing potential PCR inhibitors.

### 2.2. PCR Amplification and NGS Processing

The greatest advantage of applying NGS into diet analysis is that it can mix several PCR products, thus a large amount of data can be obtained in one NGS reaction. Multiple samples at a large scale can be analyzed in one NGS run, and as a consequence costs of diet analyses will diminish dramatically. To separate and identify samples after NGS sequencing, NGS uses incorporated tags in synthesized primers, and these tags often called MIDs can play a role to identify the individual sample [28,46]. The tagging process is completed while synthesizing the primer, which is respectively adding bases to the 5 ‘end of the forward primer and the reversed primer. The base number depends on the individual number of mixed samples, and the more samples are, the more base numbers are. But too much base numbers will affect PCR efficiency. Octamer are generally used, and the difference between octamers should be bigger than 5, which can meet the common requirement [50]. Adopting multiplex PCR can improve the efficiency of experiment, but PCR conditions need to be optimized. How to choose the DNA barcoding primers depends on the feeding habits of animals, and we summarize the frequently used primers in NGS dietary studies to facilitate the readers (Table 1). In order to suppress amplification of DNA fragments derived from the predator, a predator-specific blocking oligonucleotide is designed when preparing libraries, which can effectively improve the sensitivity in rare prey detection [51,52]. However, blocking probes can potentially block other prey species, particularly if predators and preys are phylogenetically close [53].

Mixing PCR products are pooled in equal amounts to construct an amplification library, and then NGS can be conducted. How to select NGS in order to get the sequence directly relates to the handling of subsequent data. In this case, the redundant sequence generated by PCR and NGS are mainly considered and they can be selected and removed by corresponding procedures [45,54].

### 2.3. Building a Local Reference Database

Sequences generated by the NGS platforms need to be blasted with the public and local database, and the prey species corresponding to the sequence in the database can be identified [59]. Because animals and plants vary due to geographical distributions, the public database (NCBI, EMBL, and DDBJ) just include part of DNA barcodes uploaded by local researchers, which may result into a low resolution taxa assignation [60]. In addition, there are various types of DNA barcodes, and the resolution is also different. Different DNA barcodes or DNA barcode combination can be selected according to the diet habit. But if the public database lacks of this kind of DNA barcode data, the classification accuracy of diet analysis will be reduced. This means that the local DNA barcodes database of all local potential diet resource, where animals potentially eat, should be collected and identified by both morphological and molecular methods.

The process of constructing local DNA barcode database is as follows: (1) Collecting the specimen potentially consumed by predators in the predator’s distribution, and morphologically determining the species with the assistance from the taxonomic experts; (2) Extracting the DNA and amplifying them in terms of each DNA metabarcoding marker. (3) Constructing the local reference database through Sanger sequencing. Taking analyzing diets of herbivores as an example, constructing a rbcL library can make the proportion of identification to species level reach to 72% [35]. However, if the local database is not constructed, the proportion of identification to species will be significantly reduced. For example, just 4–20% of sequences are able to be identified to species or genus level while blasting NGS data of bats [57]. The enormous DNA extracts obtained locally are therefore also considered as a resource, as new DNA barcoding regions can be amplified and sequenced based on the same DNA extracts. More importantly, the local DNA barcode database itself can be directly used in the assessment and monitoring of biodiversity [61].

### 2.4. Data Filtering and Analysis

When blasted with the established local reference database and the public database, taxon assignation of the prey can be achieved using the sequence similarity and a unique taxon will be assigned to a unique sequence, but the setting of threshold value is still controversial now. Some studies adopt the relaxed similarity threshold value to determine the taxonomic category of species, such as adopting 97% [33,44], or they may adopt more rigorous thresholds value, such as using 99% [57,62] and 100% [40]. Some researchers recommended that different threshold values should be used according to different DNA barcodes and the questions addressed [28]. For some studies that do not construct a local database, though they can adopt the clustering method to complete the differential analysis of diet composition through the Molecular operational taxonomic units (MOTU) in subsequent classification and difference analysis. Generally speaking, constructing the local database will help to improve the more accurate taxonomic assignation, with clear reference to what food resources are actually available in the habitat and ecologically meaningful to the predator [60]. For related species living in different habitats or biogeographical regions, they may share the same barcode sequence, and it may be identified to a higher taxonomic level (i.e., genus, family, phylum) when blasting a database constructed at the worldwide level.

NGS technologies have the ability to generate millions of sequence reads per sequencing run and as a consequence enormous sequence reads per sample [55]. However during this process, a variable number of erroneous sequences may originate from DNA degradation, contamination, PCR bias, primer dimers, sequencing errors, chimeras, etc. Ineffective controlling and filtering of such erroneous data can produce an overestimation of the number of molecular operational taxon units (MOTUs), and inaccurate diet assessments will interfere with the application. Generally, it is suggested to use internal controls, PCR replication and sequence distribution patterns across samples to objectively guide and choose the data filtering criteria and parameters in post-sequencing dietary data analysis.

## 3. Case Studies of NGS Based Diet Analysis

### 3.1. Investigating Effects of Animal Feeding on Environmental Changes

Diet selection plays an important role in herbivore-plant interactions, influencing both the herbivore’s population viability and plant community composition. Overgrazing poses a challenge by reducing forage quality and production, and negatively affects native plant resources. Grazing livestock diets was first assessed using universal DNA-based methods based on pyrosequencing [63]. Diet composition in two *Pyrenean chamois* populations with contrasting livestock pressure was compared with the aim to study the effect of sheep flocks on the feeding behavior [64]. Livestock depredation is the most ubiquitous type of negative interaction between humans and carnivores. With increasing livestock numbers, unprecedentedly livestock increases are sparking growing concerns over rangeland health. Meta-barcoding is a promising quantitative tool to understand resource partitioning in ungulates [65].

### 3.2. Identifying Detailed Diet Taxonomies

In dietary studies, the first that needs to be figured out is what animals eat. The diet composition of animals can be analyzed using NGS because of its high data abundance and even scarcely feeding diet can be detected because of its sensitivity. Egeter et al. [66] studied the frog species that are eaten by *Rattus norvegicus*, *Mus musculus* and *Erinaceus europaeus* utilizing NGS technology, and they found that compared with morphological microscopic analysis, NGS increased the detection rate of frogs from 2% to 70%. Jiang et al. analyzed the diet of *Cincluspallasii Temminck*, yeilding a diet covering 11 orders, 8 families and 11 genera, but nearly 50% of the sequences cannot be assigned due to lack of the local reference database. Reptiles and small mammals were found to be the main diet of the Austrian Coronella austriaca [46]. Sex-specific diet for harbor seals (*Phoca vitulina*) was identified using molecular diet analysis [67]. NGS-based DNA metabarcoding methods were considered to be very useful to provide in-depth information regarding diet profiles of the otters [51]. Detailed diet taxonomies was fully understood through a barcoding-based scat-analysis assessment in Eurasian otter (*Lutra lutra*) [68].

Diet partitioning has been developed as a strategy to avoid competition between *Ctenomys flamarioni* and *Ctenomys minutus* in the sympatric region. Soininen et al. [69] compared the wintering diets of two species of lemmings, and found that the diet composition are highly overlapped, but abundant diet resources in the study region is able to meet the demand, so the interspecific competition is not obvious. NGS-based approaches helped elucidate the complex relationship between the predator and the prey in highly speciose regions, for example, dietary resource partitioning were positively related to the ecomorphological divergence of three insective bat species [57].

Diet analysis provides a new means for studying the relationship between the cryptic species and resources and is helpful to reveal the mechanism of species coexistence and diet overlap. By enabling identification of dietary components at the species-level using NGS method, researcher found that two sympatric cryptic bat species presents significant spatial partition of foraging habitats, helping to reveal the fine-scale coexistence mechanism [70].

### 3.3. Inferring Predator-Prey-Environment Relationship

Food is the source of energy and nutrition needed for animal survival and reproduction, and diet relationships reflect the basic relationship among species. The diet varies among different predator species, and in order to avoid competition, sympatric species may evolve different foraging strategies, such as selecting different microhabitats, feeding different foods or feeding at different times [71]. Food factor plays an important role in species coexistence and species competition [72], and studying diet preference is the premise of identifying diet selection mechanism behind complex food webs. For the same species, gender difference in diet may be manifested due to different reproductive tasks undertaken by male and female individuals, and even the individual difference of feeding habit may exist due to personality [73]. By using DNA metabarcoding, dietary niche partitioning was evident among large mammalian carnivores, and livestock subsidies facilitate large-carnivore sympatry and persistence [74].

Whether animals migrate or not, when animals migrate and where animals migrate, can be environmentally induced, such as the effect of food availability [75]. The migration process needs very high energy demand, and animals adapt to the environmental change through physical, behavioral and diet changes. The microscopic identification of feces tissue showed that Pipistrellus nathusii has a higher similarity of diet between resident and migrate populations, but the NGS results with high resolution indicated that the diet difference of feeding habit exist during the two phases, inferring different foraging strategies are adopted in order to adapt to local food resource changes [62]. Assessing the variation of diet composition within a species over different life history periods, it can offer insights to comprehend the ecological niche the species occupy, as well as to infer different feeding adaptive strategies [76]. With a higher taxonomic resolution, NGS based dietary studies challenge optimal foraging theory [44]. Species with sexual dimorphism generally show a sex difference in the utilization of food resource, Kartzinel and Pringle [40] analyzed the feeding habit of Anes sagrei using NGS and found that the diet diversity of female individuals is higher than male individuals.

Habitat provides necessary food resources for animals, and unique ecological environment may evolve out specific diet selection behavior, and at the same time, the change of habitat can lead to changes of food availability and diet diversity. The study of the relationship between feeding habit and habitat provides a basis for understanding animals’ feeding strategies and habitat selection. NGS can efficiently analyze the feeding habit of animals locating in different habitats on larger spatial scales. Clare, Symondson [28] analyzed the feeding habit of browntail bats in different habitats using NGS, and the result shows that bats in slightly polluted habitats have more abundant food, and they proposed that the food quality of bats can be regarded as an indicator to evaluate environmental quality. Trevelline, Latta [77] conducted the feeding habit analysis of Parkesia motacilla using NGS, and they found that terrestrial insects have a large proportion of Parkesia motacilla, which changes the previous view that Parkesia motacilla prefers to insects living in polluted aquatic environment.

Ecologists always pay more attention to the relationship and interaction between predators, prey and environment. Herbivores may have a close relationship with the formation and distribution of natural plant communities, and even have influence on alien invasive plants. The feeding preference of *Odocoileus virginianus* was investigated on native plants and alien invasive plants using NGS [35], which found that local plants were consumed as their main diet, leading to the expansion of alien invasion plant to a certain extent. The feeding habit of three types of rodents provides a scientific basis for the biological prevention of rats’ damage [7].

### 3.4. Ecosystem Monitoring

For the species of generalist feeding or wide distribution, they are biological indicator species, because they rank as the abundant top-level consumers in the food webs, and their diet composition can be used to infer overall ecosystem status [78]. Avian endoparasite communities can be investigated by using metabarcoding approach among birds [79]. Hidden biodiversity can be discovered through the use of complementary monitoring of fish diet based on DNA barcoding, which is helpful to understand the complex ecosystem functions [80]. An overall biodiversity monitoring was evaluated by analyzing the quantitative trophic interactions among sympatric carnivores from three assemblages in the Mountains of Southwest China [74].

## 4. Current Limitations

### 4.1. Technical Errors

Despite its power, NGS-based diet analysis as developed has several limitations associated with technical errors, which can distort the signal of taxonomic composition and diversity [31]. One obvious limitation is its current dependency on sequencing PCR amplification products, and diet analysis based on amplicon sequencing are more severely influenced by biases than more common NGS applications such as resequencing of genes, transcriptomes or genome [81]. PCR can introduce errors in the NGS-based diet data, with three error origins: degraded DNA template, errors during amplification and errors during sequencing [45]. The substances derived from prey digestion significantly reduce DNA amplification success rates in fecal or stomach diet samples. Even using highly conserved DNA-barcoding primers, DNA degradation rate and PCR inhibitors in dietary samples influence detection of prey DNA, and certain DNA fragments show preferential amplification efficiency and primer competition occur in a multiplex PCR [39]. Thus, prior optimization of PCR protocols to improve primer efficiency and minimize PCR errors still remains in mind as the first importance. It is highly recommended the proofreading taq polymerase be used to produce PCR amplicons with fewer bias in future NGS-based diet analysis [82].

Contamination is another non-neglected error source of diet analysis using NGS. The trace amount of prey DNA from different dietary samples, combined with extremely high sensitivity of PCR step and NGS technology, indicates even minor contamination will make it inevitable in the subsequent dietary data, regardless of strict conformance with good laboratory performance for minimizing the risk of contamination especially when universal primers are used, which will result in overestimation of diet diversity or the misleading results. Contamination in NGS-based diet analysis mainly originates in two forms: physical and ecological. Physical contamination may be randomly from single DNA molecules present in the lab room, DNA extraction tubes during PCR conduction, contaminated reagents or pipetting errors, which necessitates physical separation of experimental equipments used in pre and post-PCR amplification steps. To further minimize appearance of contaminants, Valentini et al. [47] recommended reducing the cycles during PCR (<35 cycles).

### 4.2. Biological Factors

Dietary items occasionally consumed can be identified in the case that secondary predation occurs where a predator consumes another type of predator that contains some prey in its feces [40,60]. For example, marine species may accidentally eat planktonic food, and contaminant monitor studies will need to be taken into account, which plays a greater role for benthic and intertidal pathways in trophic webs [83]. Some NGS-based diet analysis encountered a higher rate of presumed fungal and bacterial contamination and greater amplification of predator DNA [28,49]. Taxon-specific primers need to be further developed and evaluated to improve the specificity and efficiency of capturing prey DNA in dietary samples, instead of detecting bacterial and fungal contamination or even host DNA [84].

NGS-based diet analysis allows us to establish and evaluate species-level taxonomic assignments and diet assessments, instead of traditional ordinal-level assessments obtained from morphological analysis. Dietary pattern could be different between at the ordinal and the species, which is largely due to saturation of the ordinal-level data, while data at the species-level have not yielded a plateau [28]. However, the taxonomic resolution achieved is still limited by variability of the selected marker and linked to the availability of the local reference database. Higher resolution power in identifying more prey species can be accomplished by building the local DNA-barcoding database or using additional specific markers. Short size primers may reduce taxonomic resolution power in some cases but can increase the likelihood of amplication of degraded DNA. The combined approach of multiple primers targeting different regions will provide the highest taxonomic resolution, such as trnL, rbcL, matK or ITS [32,38,39,60], when studying the diet of herbivores species. The analysis of MOTUs can provide higher resolution information of the sequence assignment and diet diversity, which is useful for estimating foraging habitat overlap in order to infer the pattern of resource partitioning where the identification at the species is not necessary.

Compared with a descriptive list of prey species and diet diversity, the proportion of animals’ ingested items in food webs and diet preferences are the key focus of animal ecologists. However, it is still fraught with problems that many different studies have shown that quantitatively analysis is difficult in amplicon sequencing [60]. Whether the relative sequence counts generated by NGS quantitatively reflects the prey items consumed remains controversial [81]. The control experiment of feeding 4 kinds of fish to pheasant find that the NGS data are relatively accordant with the data calculated by parallel quantitative PCR, which indicates that the sequencing number of NGS can quantitatively reflect the ingesting quantity [85]. However, studies about Phoca vitulina indicate that the proportion of feeding fish is not accordant with the sequencing number calculated by NGS [81]. Reasons causing this bias include biological factors and technical factors. Bias of technically obtaining quantitative data may appear during DNA extraction, DNA pooling, biased amplification, primer tags, sequencing direction and bioinformatic parameter settings. Beyond the technical problems, some biological features may cause noise information and interfere with the quantitative signals. Biological factors include several aspects as followings: DNA copy is different in different tissue cells; different diet tissues contain different number of cells; animal’s digestibility variability for each diet item.

## 5. Conclusions and Future Developments

There are more important and meaningful ecological questions where NGS-based diet analysis may support the insights than we expect, from individual diet items and preference to the evolutionary causes and ecological consequences of variations in ecological studies. Reviews in this scopic always highlight the need that more and better laboratory and field work are planned, for better understanding the factors influencing dietary spatio-temporal variations among individuals or species. This review is either no exception, as such dietary studies are still as important as they were just like when one of the first innovative calls appeared.

Given the existence of uncertainties in NGS-based diet analysis and quantitative interpretation, then how can the technical data of NGS be qualitatively and quantitatively reliable and credible? In the same study, trying another diet analysis method at the same time is an effective way to verify the accuracy of data. Srivathsan et al. [86] adopted the “metagenomics” approach to quantitatively analyze the diet of Pygathrix nemaeus and this method does not need PCR before transporting to the NGS platform, and they found that metagenomics has the advantage of yielding more precise plant identifications than amplification sequencing approach. However, in another study Odocoileus virginianus, contrary results were obtained, indicating amplification sequencing method has a higher accuracy [35].

With the gradual maturity and stability of NGS technology and the attention and control of error factors, NGS-based diet analysis will be adopted more widely and deeply [31]. At present, the vast majority animals’ diet in the world is still in a rougher qualitative description stage. With the cost reduction of NGS technology, we believe that more researchers can utilize this technology into diet analysis and explain more complex but meaningful problems by integrating diet results. For example, exploring the relationship between ingesting plants and ecological processes of plant pollination and seed diffusion in order to determine how a specific animal mediate the pollinating of a specific plant, and to position the ecological role of animals in food web structure [87]; Studying the impact of climate change on animals’ diet preferences will benefit predicting how the animals’ preferring diet will change with climate change and simulating how animals respond to global warming by changing diet [54,88]; Combining NGS technology with other technical methods will develop a border space for the application of diet analysis. NGS has a unique advantage in determining types and differences of diet, and stable isotope analysis can analyze the source and energy flow of diet, indicating different methods can complement with each other and have important insights for studying complex food webs [89,90,91]. Combined with NGS technology, nutrient geometry can be used to explore animals’ nutrient demand and foraging strategies, and investigate how animals respond to forage resources to achieve a specific ratio of two or more nutrients in the diet [4]. Integrated chemical and isotopic analysis of ceramic sherds from Pastoral Neolithic archaeological contexts in Kenya and Tanzania reveals a history reliance on milk, meat, and plant for ancient herding societies in eastern Africa [92], and the application of molecular diet analysis is useful to identify their precision feeding. Metagenomics is applied to effectively characterize the diet of herbivores, particularly when a localized reference database is not available for minibarcodes used in metabarcoding [93]. With the advance of omics, the field of nutrigenomics spans multiple disciplines and includes the effects of diet on genome stability, epigenome changes, RNA and micro-RNA expression (transcriptomics), protein expression (proteomics), and changes in metabolite levels (metabolomics) [94,95].

## Figures and Tables

**Table 1:** Available versatile DNA barcoding primers for specific preys used in previous studies based on next generation sequencing based diet analysis.

Prey Types	Prey Taxa	Target	Primer Name	Primer Sequence (5′–3′)	Blocking Nucleotides	References
Vertebrata	Metazoan	COI	mlCOIint-F:	GGWACWGGWTGAACWGTWTAYCCYCC	Human blocking: CTATGCTTAGCCCTAAACCTCAACAGTTAAATCAACAAAACTGCT-C3 Mammal blocking: CTAGGGATAACAGCGCAATCCTATT-C3 or GATAGCTTACATAACAAAACTATCTGC-C3	[55]
			jgHCO2198-R:	TAIACYTCIGGRTGICCRAARAAYCA		
	Mammal	12S V5	12SV5-F:	TTAGATACCCCACTATGC		[39,45]
			12SV5-R:	TAGAACAGGCTCCTCTAG		
	Amphibian	Cytb	RT-F	TACAGCCGATACCTCCCTC		[46,51]
			RT-R	TTCATGTCTCTTTGTAGAGG		
	Fish	16S	Chord_16S_F	CGAGAAGACCCTRTGGAGCT		[42,56]
			Chord16S_R	CCTNGGTCGCCCCAAC		
Invertebrate	Arthropoda	COI	ZBJ-Art-F	AGATATTGGAACWTTATATTTTATTTTTGG		[28,57]
			ZBJ-Art-R	WACTAATCAATTWCCAAATCCTCC		
	Arthropoda	16S	IN16STK-F	TGAACTCAGATCATGTAA		[40]
			IN16STK-R	TTAGGGATAACAGCGTAA		
	Mollusca	16S	16SMAV-F	CCAACATCGAGGTCRYAA		[39]
			16SMAV-R	ARTTACYNTAGGGATAACAG		
	Annelida	12S	185F	TGTGTACTGCCGTCGTAAGCA		[46]
			14233R	AAGAGCGACGGGCGATGTGT		
Plant	Universal	trnL P6	G	GGGCAATCCTGAGCCAA		[38,58]
			H	CCATTGAGTCTCTGCACCTATC		
	Universal	rbcL	rbcLa-F	ATGTCACCACAAACAGAGACTAAAGCGTAAAATCAAGTCCACCRCG		[32]
			rbcLa-R			
	Universal	rbcL	rbcL-F	CTTACCAGYCTTGATCGTTACAAAGGGTAAAATCAAGTCCACCRCG		[35]
			rbcL-R			

## Data Availability

Not applicable.
